# Mouse Model of Weak Depression Exhibiting Suppressed cAMP Signaling in the Amygdala, Lower Lipid Catabolism in Liver, and Correlated Gut Microbiota

**DOI:** 10.3389/fnbeh.2022.841450

**Published:** 2022-05-19

**Authors:** Kousuke Shimada, Masakatsu Nohara, Akihito Yasuoka, Asuka Kamei, Fumika Shinozaki, Kaori Kondo, Ryo Inoue, Takashi Kondo, Keiko Abe

**Affiliations:** ^1^Group for Food Functionality Assessment, Kanagawa Institute of Industrial Science and Technology, Kawasaki, Japan; ^2^Department of Applied Biological Chemistry, Graduate School of Agricultural and Life Sciences, The University of Tokyo, Tokyo, Japan; ^3^Division of Disease Systems Biology, RIKEN Center for Integrative Medical Sciences, Yokohama, Japan; ^4^Laboratory of Animal Science, Kyoto Prefectural University, Kyoto, Japan

**Keywords:** depression, microbiota, nicotinamide adenine dinucleotide, amygdala, corticosterone, cyclic adenosine monophosphate (cAMP)

## Abstract

To establish a mouse model of weak depression, we raised 6-week-old C57BL/6N mice in single (SH) or group housing (GH) conditions for 2 weeks. The SH group showed less social interaction with stranger mice, learning disability in behavioral tests, and lower plasma corticosterone levels. The cecal microbiota of the SH group showed significant segregation from the GH group in the principal coordinate analysis (PCoA). Transcriptome analysis of the amygdala and liver detected multiple differentially expressed genes (DEGs). In the amygdala of SH mice, suppression of the cyclic adenine monophosphate (cAMP) signal was predicted and confirmed by the reduced immunoreactivity of phosphorylated cAMP-responsive element-binding protein. In the liver of SH mice, downregulation of beta-oxidation was predicted. Interestingly, the expression levels of over 100 DEGs showed a significant correlation with the occupancy of two bacterial genera, *Lactobacillus* (Lactobacillaceae) and *Anaerostipes* (Lachnospiraceae). These bacteria-correlated DEGs included JunB, the downstream component of cAMP signaling in the amygdala, and carnitine palmitoyltransferase 1A (Cpt1a), a key enzyme of beta-oxidation in the liver. This trans-omical analysis also suggested that nicotinamide adenine dinucleotide (NAD) synthesis in the liver may be linked to the occupancy of *Lactobacillus* through the regulation of nicotinamide phosphoribosyltransferase (NAMPT) and kynureninase (KYNU) genes. Our results suggested that SH condition along with the presence of correlated bacteria species causes weak depression phenotype in young mice and provides a suitable model to study food ingredient that is able to cure weak depression.

## Introduction

Young stage social stress affects an individual’s mental health and is recognized as one of the risk factors of depression in humans (Lupien et al., 2009; Walker et al., 2019). Several animal models, such as the social defeat mode and maternal deprivation model, have been developed to explore their effects (Bian et al., 2015; Yang et al., 2016; Amini-Khoei et al., 2019; Oizumi et al., 2019; Sato et al., 2019). In these models, animals were exposed to stresses that induce hypothalamic-pituitary-adrenal (HPA) axis activation and acute increment of corticoid. It has been proposed that this stress cycle may cause excessive adaptation of the HPA axis and the depression status with low corticoid levels (Keller et al., 2017). With reference to the neuronal response to stressors, cyclic adenosine monophosphate (cAMP) signaling has been proposed to play an essential role in the amygdala. By the use of cAMP analog injection or phosphodiesterase (PDE) gene knock out, it has been shown that activation of cAMP signaling in the amygdala was necessary for fear memory consolidation by the amygdala (Viosca et al., 2009; Tronson et al., 2012; Cowansage et al., 2013; Ranjan et al., 2015). Corticotropin-releasing hormone is one of the candidates for extra-cellular stimuli that trigger cAMP signaling, but several neurotransmitters, such as noradrenaline, are also considered to be involved (Keil et al., 2016). However, these depression models may be too severe to study relatively weak depression that afflicts a substantial proportion of the population in modern society (Cho et al., 2019). During the lactation period, animals depend on significant biological inputs from their mothers. In a previous study, the offspring of a germ-free mother exhibited lower body weight and less locomotor activity at a mature stage, and these phenotypes could be restored by fostering with a normal mother (Tochitani et al., 2016). Benner et al. (2014) isolated pups from their mothers for 3 h per day and cohoused them with the control siblings until they were 12 weeks old. The deprived mice exhibited a lower score in reversal learning tasks in their adult stages. These results suggest that both microbiological and physical interaction with the mothers are critical for maintaining the offspring’s mental health. There are also studies about the effects of the social environment at the young adult stage. It is reported that single housing (SH) of 7-week-old mice for 3 days caused a decrease in urine corticosterone levels and the value remained lower than the group-housed mice for 3 weeks (Kamakura et al., 2016). Ieraci et al. (2016) maintained 8-week-old mice in SH condition for 4 weeks and found that the mice moved more distance than the group-housed mice in the open-field test and exhibited lower corticosterone levels. These results indicate that the decrease in social interaction during the young adult stage causes depression-related phenotypes. However, these animal studies provide little information about intrinsic molecular changes, such as altered tissue transcriptome, microbiome, and their mutual interaction. There have been attempts to cure depression by food ingredients, such as polyphenols, pre- and probiotics (Hurley et al., 2014; Wei et al., 2019; Chen et al., 2021). However, the animal models used in these studies did not necessarily reflect the status of modern human society, and a more suitable model has been needed to study food-based therapeutics. Here, we present a mouse model of depression that shows weaker phenotypes than models reported previously. We also conducted correlational analysis between multi-tissue transcriptomes and the gut microbiome that enabled us to evaluate the homeostatic contribution of microbiota to the brain and liver functions in weak depression. Our new animal model provides a basis for future studies exploring treatment against weak depression, especially by the use of functional food ingredients.

## Materials and Methods

### Mouse Maintenance

**FIGURE 1:**
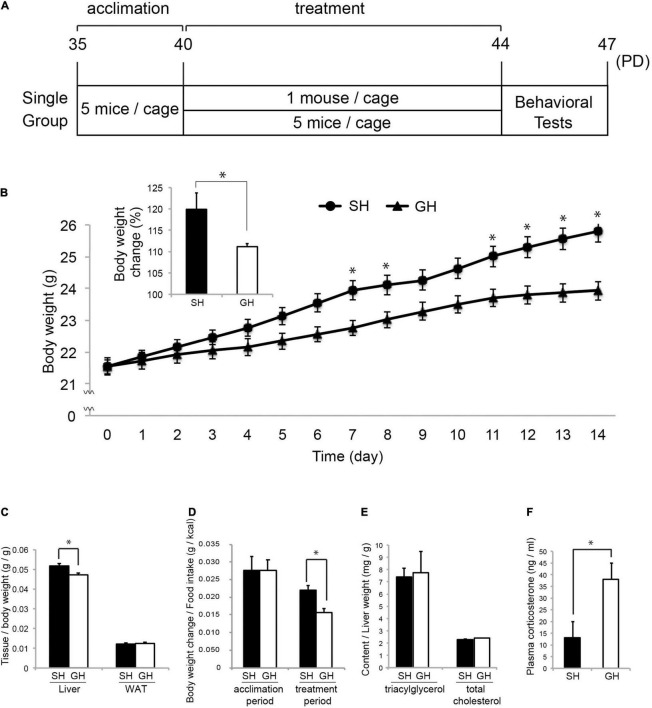
Mouse housing and effect on the physical parameters. **(A)** Five-week-old (PD35) male C57BL/6N mice were acclimated for 5 days in group housing (GH) condition and maintained in single (SH) or GH conditions for 14 days. They were kept in the same open air rack to equalize all physical conditions other than social interaction and handled separately to reduce physical interaction between individuals (*Materials and Methods*). On the next day, the mice were subjected to the behavioral test in the following order: open-field test, Y-maze test, novel object recognition test, and social interaction test. Mice designated for tissue sampling were not tested. **(B)** Body weight (BW) change during the experimental period (from PD40 to PD44) with the inset of percentage BW change (*n* = 35 for each group). **(C)** Relative weight of the liver and epididymal white adipose tissue (WAT) at the time of sacrifice, PD44 (*n* = 25 for SH and GH). **(D)** Assimilation quotient of each group calculated as the ratio of the BW increase to the food intake (kcal) during the acclimation (*n* = 15 for each group) or the experimental period (*n* = 35 for each group). **(E)** Triacylglycerol (TG) and total-cholesterol (TCHO) content of the liver (*n* = 4 for each group). **(F)** Comparison of plasma corticosterone levels (*n* = 10 for each group). Values are mean ± SEM. *Significant difference detected by unpaired *t*-test (*p* < 0.05).

Five-week-old (postnatal day 35, PD35) male C57BL/6N mice were procured from Charles River (Hino, Japan). The animals were shipped in cardboard cages (5 mice/1 cage), housed in air-conditioned rooms (22 ± 2°C, and 50 ± 5% humidity), and subjected to a cyclic 12 h light (8 a.m.–8 p.m.) and dark (8 p.m.–8 a.m.) environment. The light intensity was 100 lx at the front of the cages and 40 lx behind the cages. Five mice were acclimatized in a cage (24 cm length × 17.2 cm width × 12.9 cm depth, 413 cm^2^ floor area) with free access to the chow diet (MF, Oriental yeast, Tokyo, Japan) and water until PD40 (Figure 1). Subsequently, mice were shifted to the SH condition (one mouse in one cage of 18 cm length × 11 cm width × 11 cm depth, 198 cm^2^ floor area) or in the group housing (GH) condition (same as the acclimatization) with free access to the chow diet and water until PD44. Mice and cecal samples in the different conditions were handled separately under sterile conditions. Cecal content was collected on the PD44, and the mice were euthanized by cervical dislocation. The amygdala, hypothalamus, and liver were collected and stored at −80°C. Mice intended for behavioral analysis were raised under SH or GH conditions and subjected to the behavior tests that included the open-field test on PD44, Y-maze test on PD45, novel object recognition test on PD46, and social interaction test on PD47. Body weight was regularly measured every day. The food intake was measured at the same time. For the calculation of assimilation ratio, the total body weight increment from PD35 to PD40 (the acclimation period) or from PD40 to PD44 (experimental period) was divided by the total food intake in each period. The behavioral tests that are listed below were performed under the same maintaining condition. All procedures were performed in accordance with the Ministry of Education, Culture, Sports, Science, and Technology guidelines for the use of experimental animals. The animal experimental protocol was in accordance with Animal Research: Reporting of *In Vivo* Experiments (ARRIVE) guidelines and approved by the animal committee of the Innovation Center of NanoMedicine (Kawasaki, Kanagawa, Japan).

### Behavioral Tests

Social interaction test: Mice were placed at the center of the field for the novel object test where an empty meshed box was placed (Supplementary Figure 1A). The movement of the mice was observed for 5 min (exposing period) by a camera (Cannon iVIS HFR62, 24 frame/s, 1,280 × 720 pixels, 4,000 kbps, MP4) situated 170 cm above the center of the field. The mice were then placed in an empty cage for 2 min and returned to the center of the field where a stranger male mouse in a meshed box was placed. The movement of the mice was observed for 5 min (testing period). The stranger mice were 8-week-old male C57BL/6N mice and were selected from the other cages in the same animal room. For this analysis, we defined the nine square zones according to their proximity to the meshed box containing the stranger mouse and two square zones (10 cm × 10 cm) at the other corner of the field. Spending time and stopping time of head point in each zone from 10 s to 5 min 10 s (exposing period and testing period) were analyzed using the Smart 3.0 Video-Tracking System (Panlab Harvard Apparatus).

Novel object recognition test: Mice were placed at the center of the field (40 cm × 40 cm width × 47 cm depth) where two identical objects were placed (object A, Supplementary Figure 1C). The movement of the mice was recorded for 10 min as described for the social interaction test (exposing period). Mice were then placed in an empty cage for 2 min and returned to the center of the field where two different objects were placed (object B, Supplementary Figure 1C), and their movement was recorded for 5 min (testing period). We defined the head contact of mice with the objects as their proximity under 1.5 cm. The preference ratio was calculated based on the contact time to the objects. Movement of the mice between 1 and 10 min (exposing period) or 10 s and 5 min 10 s (testing period) was analyzed by Smart 3.0 software. The mice that showed no contact with the objects were not analyzed.

Y-maze test: Mice were placed at the end of a Y-maze (3 cm width × 40 cm length arms, 12 cm wall height, Supplementary Figure 1D) illuminated at 250–300 lx. The movement of the mice was noted for 10 min in the same way as the social interaction test, and the total entry counts and spontaneous alternation (entry to the different arms, i.e., arm ABC = 1, arm ACA = 0, and arm ABCBCBCACB = 3) were analyzed by visual inspection from 0 to 10 min.

Open-field test: Mice were placed at the center of an open field (75 cm × 75 cm width × 37.5 cm depth, Supplementary Figure 1E) that was made with white plastic and illuminated at 250–300 lx. The movement of the mice was observed for 10 min in the same way as the social interaction test. Moving distance, spending time, and velocity in the center zone (30 cm × 30 cm at the center) or in the other zone (peripheral zone) were analyzed using the Smart 3.0 software from 1 to 10 min.

### RNA-Seq Analysis

Total RNA was isolated from the amygdala, hypothalamus, or liver using TRI Reagent (MRC molecular research center, OH, United States). Total RNA from the liver was further purified using a SimplyRNA Tissue Kit on the Maxwell RNA extraction system (Promega). RNA quality was accessed using RNA 6000 Nano Series II Kit on Agilent 2100 Bioanalyzer (Agilent141 Technologies, Santa Clara, CA, United States). RNA integrity number for each tissue RNA was higher than 8.5. In total, 200 ng of Total RNA was constructed in a library using Smarter Stranded Total RNA Sample Prep Kit HI Mammalian (Clontech Laboratory, Inc.). The library was quantified using a High Sensitivity DNA kit on Agilent 2100 Bioanalyzer and subjected to 50 bp single-end sequencing analysis (55.7 million/sample on average) on a Hiseq System (Illumina). The FASTQ data were checked by the FastQC quality check program and trimmed using the fastx-clipper and cutadapt program to remove adaptor sequence (>1%) and low-quality reads (quality < 30, length < 20 bases). Cleaned reads were mapped against Mus musculus reference genome GRCm 38 (mm10^1^) using TopHat program (library-type FR second Strand -g20 -N2). The rate of mapped reads to the obtained reads was 96.5% on average. The resulting binary alignment map (BAM) data were used to obtain the reads on the exons of known protein-coding genes (mm10) on the featureCounts program^2^. The reads per kilobase of exon model per million mapped reads (RPKM) for each tissue were filtered (RPKM < 0.3). The rate of filtered reads to the obtained reads was 56.8% on average. Differentially expressed genes (DEGs) were predicted using the DESeq2 package on R statistical software (*p* < 0.01)^3^. Cluster dendrograms were drawn by the hclust package^4^.

### Ingenuity Pathway Analysis

Differentially expressed genes were subjected to QIAGEN’s Ingenuity Pathway Analysis (IPA, QIAGEN Redwood City^5^). Upstream analysis was used to predict the cellular or endocrine signals that may have been affected by a SH condition. The differential expression of the genes was coded as + 1 (SH > GH) or − 1 (SH < GH), and the regulation of the signals was deduced as positive or negative z-scores. Only those with absolute z-score larger than 2.5 and with values of *p* less than 0.01 were selected as significantly regulated upstream regulators.

### Microbiome Analysis

DNA was extracted from the cecal samples using the bead-beating method described by Odamaki et al. (2016). The 16S rRNA V3–V4 region was amplified using Tru357F (5′-CGCTCTTCCGATCTCTG TACGGRAGGCAGCAG-3′) and Tru806R (5′-CGCTCTTCCGATCTGAC GGACTACHVGGGT WTCTAAT-3′) primers and sequenced on Illumina MiSeq. The processing of sequencing data, such as quality filtering, chimera check, operational taxonomic unit (OTU) definition, and taxonomy assignment, was carried out using the Quantitative Insights Into Microbial Ecology (QIIME software package version 1.8.0.), EA-Utils (ver. 1.1.2–537), the gold database^6^, and USEARCH (ver. 5.2.32^7^). Principal coordinate analysis (PCoA), alpha diversity (Shannon index) analysis, and beta diversity analysis were conducted using vegan (Package vegan 2015, version 2.3–4) on R (2015, version 3.1.3) (Oksanen et al., 2016). All data sets (*n* = 15) were used for between-group comparison (Figure 3B–D and Supplementary Table 1). A part of the data (*n* = 4) was used for correlational analyses (Figures 3E,F and Tables 1–3).

**TABLE 1:** The functional enrichment analysis of the genes affected by single housing (SH) condition in the amygdala.

Tissue	Upstream Regulator	Description	Predicted Activation State	Activation z-score	*p*-value of overlap	Gene regulation	Bacteria correlated	No correlation
Amygdala	FADD	Fas Associated *Via* Death Domain	Activated	2.24	3.51.E-04	SH up		Elavl2
						SH down	Junb (*Lactobacillus*+)	Egr1, Fos, Gadd45b
	Hdac	Histone Deacetylase	Activated	2.20	6.28.E-03	SH up		
						SH down		Arc, Egr1, Fos, Gadd45b, Nr4a1
	CREM	cAMP Responsive Element Modulator	Inhibited	−2.75	1.32.E-10	SH up		Mns1
						SH down	Junb (*Lactobacillus*+), Per1 (*Anaerostipes*−)	Arc, Ciart, Csrnp1, Egr1, Fos, Gadd45b, Irak1, Nr4a1, Sik1
	CREB1	cAMP Responsive Element Binding Protein 1	Inhibited	−2.75	8.37.E-08	SH up		Mns1
						SH down	Junb (*Lactobacillus*+), Per1 (*Anaerostipes*−)	Arc, Ciart, Csrnp1, Egr1, Fos, Gadd45b, Nr4a1, Sik1
	FOXO3	Forkhead Box O3	Inhibited	−2.41	2.80.E-03	SH up		Itgam, Lcp2
						SH down	Junb (*Lactobacillus*+)	Egr1, Fos, Gadd45b
	MAPK3	Mitogen-Activated Protein Kinase 3	Inhibited	−2.22	2.53.E-05	SH up		
						SH down	Junb (*Lactobacillus*+)	Arc, Egr1, Fos, Nr4a1
	Pkc(s)	Protein Kinase C	Inhibited	−2.21	5.38.E-08	SH up		Hsp90b1, Hspa5, S100a1
						SH down	Junb (*Lactobacillus*+), Per1 (*Anaerostipes*−)	Dbp, Egr1, Fos, Gadd45b, Nr1d1 (Rev-ErbA)
	PSEN1	Presenilin 1	Inhibited	−2.18	1.58.E-10	SH up	Uqcrh (*Anaerostipes*+)	Atp5e, Canx, Ccr5, Fbxw7, Hsp90aa1, Hspa5, Hspa8, Pea15a
						SH down	Per3 (*Anaerostipes*−), Slc11a2 (*Anaerostipes*−)	Arc, Dbp, Fos, Fzd4, Gadd45b, Hpca, Mthfd1l, Slc38a2
	NR3C2	Nuclear Receptor Subfamily 3 Group C Member 2 (aldosterone receptor)	Inhibited	−2.00	2.84.E-03	SH up		Fcrls, Fkbp4
						SH down	Junb (*Lactobacillus*+)	Cacna1h, Egr1
	Pka	cyclic dependent protein kinase	Inhibited	−2.00	3.32.E-03	SH up		
						SH down	Junb (*Lactobacillus*+)	Egr1, Fos, Nr4a1

*Shaded columns mark the upstream regulators related to cyclic adenine monophosphate (cAMP) signal.*

### Correlational Analysis

In total, 8 mice (4 from each experimental group × 2) were examined for correlation between the relative abundance of cecal bacteria at the genus level and the gene expression in each tissue. The gene expression levels were obtained by the size factor normalization of raw count data using the DESeq2 package on R statistical software. Bacteria occupancies were obtained by dividing annotated reads by total reads, and those not detected in more than 2 mice from each experimental group were not analyzed. Pearson’s correlation coefficient (>0.92) and values of *p* (<0.01) were used to select the genes detected in each tissue.

### Biochemical Analysis

The liver triacylglycerol (TG) and cholesterol levels were analyzed by Skylight Biotech (Akita, Japan^8^). The corticosterone levels were measured following the procedure described by Kanesaka et al. (1992). In brief, mice were euthanized by cervical dislocation, and the trunk blood was collected in sampling tubes containing heparin (10 IU/sample). Plasma was prepared by centrifugation, extracted by diethyl ether, and subjected to the quantification of corticosterone using Corticosterone ELISA Kit (Cayman Chemical Company, MI, United States).

### Immunohistochemistry

Mice were raised as described above, euthanized by cervical dislocation, and whole brains were isolated manually, trimmed, and fixed in 10% neutralized formaldehyde (062-01661, Wako) at 4°C for 16 h. Subsequently, hemispheres were dehydrated, embedded in paraffin (TissuePrep, melting point 56–57°, Fisher Scientific), and sectioned by 3 mic-m steps on RETRATOME REM710 (Yamato Kogyo, Japan). Then, sections were deparaffinized, soaked in the antigen retrieval solution (HistoVT One; Nacalai Tesque, Kyoto, Japan) at 100°C for 20 min, and blocked by 2.5% normal horse serum in 1 × tris-buffered saline (TBS). Subsequently, they were reacted with anti-pcAMP-responsive element-binding protein (CREB) antibody (#9198, Cell Signaling) or anti-CREB antibody (#9197, Cell Signaling) diluted (1/2,500) in 1 × TBS containing 1% bovine serum albumin (BSA) at 4°C for 16 h. The sections were further rinsed twice in TBS containing 0.01% Tween20 (TBS-t) and reacted with secondary antibody (Histofine Simple Stain MAX PO (R), Nichirei, Japan) diluted (1/5) in 1 × TBS containing 1% BSA at 22°C for 30 min. The sections were rinsed twice in TBS-t and the signal was developed using TSA Plus Fluorescein in the diluent (1/200, PerkinElmer) at 22°C for 2 min. The sections were then rinsed twice in TBS-t stained with 4’,6-diamidino-2-phenylindole (DAPI) solution (1/2,000, DOJINDO) at 22°C for 10 min and twice in TBS and enclosed in Immu-Mount (Thermo). The sections were observed under a fluorescence microscope/camera system (DP73-SET-A and IX71N, Olympus), and the images were analyzed by NIH image.

### Region of Interest and Cell Counting

The region of interest (ROI) was established as a 675 × 675 pixel box in the images of the medial amygdala in Bregma −1.58 to −1.98 (Paxinos and Franklin’s the Mouse Brain in Stereotaxic Coordinates) (Paxinos and Franklin, 2001). Sections (4 sections/one mouse) were prepared from 3 to 4 mice in each experimental group. The images of 2–4 ROIs/individuals were taken and the numbers of pCREB or CREB signals were calculated using the threshold value of T20 on the NIH image. DAPI signals were calculated in the same way using the threshold value of T45. The number of pCREB or CREB signals was divided by that of the DAPI signal in each ROI.

### Statistical Analysis

All data are presented as mean ± SEM. For physical and biochemical analyses, data were tested by unpaired Student’s *t*-test (*p* < 0.05). For behavioral analysis, data were tested by Kruskal-Wallis test (*p* < 0.05).

## Results

### Effect of Short-Term Single Housing on Physical Parameters and Behavior of Young Mice

**FIGURE 2:**
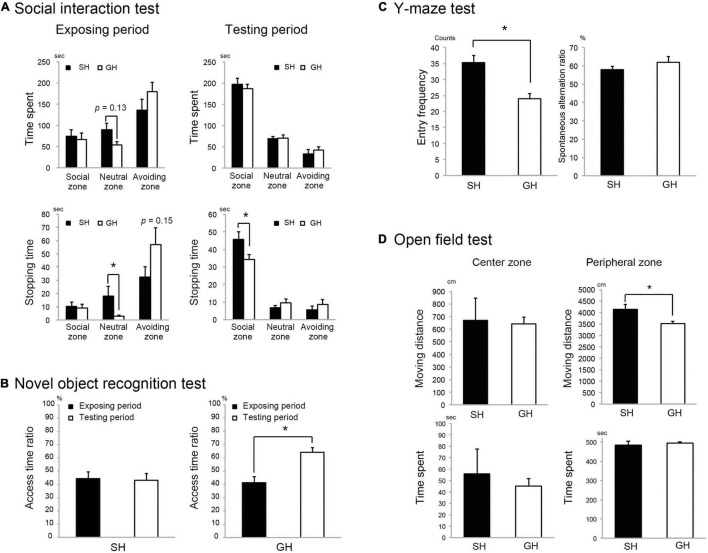
Effect of housing condition on mice behavior. **(A)** Single housing (SH) mice stopped longer in the neutral zone and in the social zone (Supplementary Figure 1A) than group housing (GH) mice during the exposure and the testing period, respectively (*n* = 10 for each group). **(B)** SH group mice showed less interest to access the novel object than GH mice (*n* = 7 for each group). **(C)** SH mice showed a larger arm entry frequency than GH mice in the Y-maze test (*n* = 10 for each group). **(D)** SH mice moved significantly longer distance than the GH mice (*n* = 10 for each group) in the peripheral zone of the open field (Supplementary Figure 1E). Values are mean ± SEM. *Significant difference detected by Kruskal-Wallis test (*p* < 0.05).

Six-week-old male C57BL/6N mice were raised in the SH or GH condition for 2 weeks (SH or GH mice) and subjected to the biochemical or the behavioral analyses (Figure 1). We chose male mice to avoid the effect of the menstrual cycle on the experiment. SH mice gained more body weight and liver weight per body weight than GH mice (Figures 1B,C) and exhibited a higher assimilation quotient (Figure 1D). The TG and the total cholesterol (TCHO) content in the liver showed no significant difference between the two groups (Figure 1E). It was notable that SH mice showed a lower level of plasma corticosterone than GH mice (Figure 1F). Next, we compared behavioral parameters between SH and GH mice. In the social interaction test (Figure 2A), no significant differences were observed in spending time in all defined zones between SH and GH mice during the testing period (Social, Neutral, and Avoiding zones in Supplementary Figure 1A). However, there were differences in stopping time in the Neutral zone during the exposure period and in the Social zone, during the testing period although no significant differences were observed in stopping time in each subdivided area (Supplementary Figure 1B). In the novel object recognition test (Figure 2B), SH mice showed no difference in access time ratio between the familiar object and the novel object while GH mice showed higher access to the novel object. The decline of social interaction with the newcomer mice and the decreased learning ability of novel objects can be classified as depressive behavior (Reincke and Hanganu-Opatz, 2017; Endo et al., 2018). SH mice showed higher entry frequency than GH mice in the Y-maze test (Figure 2C) and moved longer distance than GH mice in the Peripheral zone of the open field (Figure 2D). This kind of hyperactivity is reported to be one of the spectra of depression (Hu et al., 2016; Nickel et al., 2019). Similar behavior has been observed in the maternal deprivation mouse model (Amini-Khoei et al., 2019).

### Analysis of Tissue Transcriptomes and Cecal Microbiome

**FIGURE 3:**
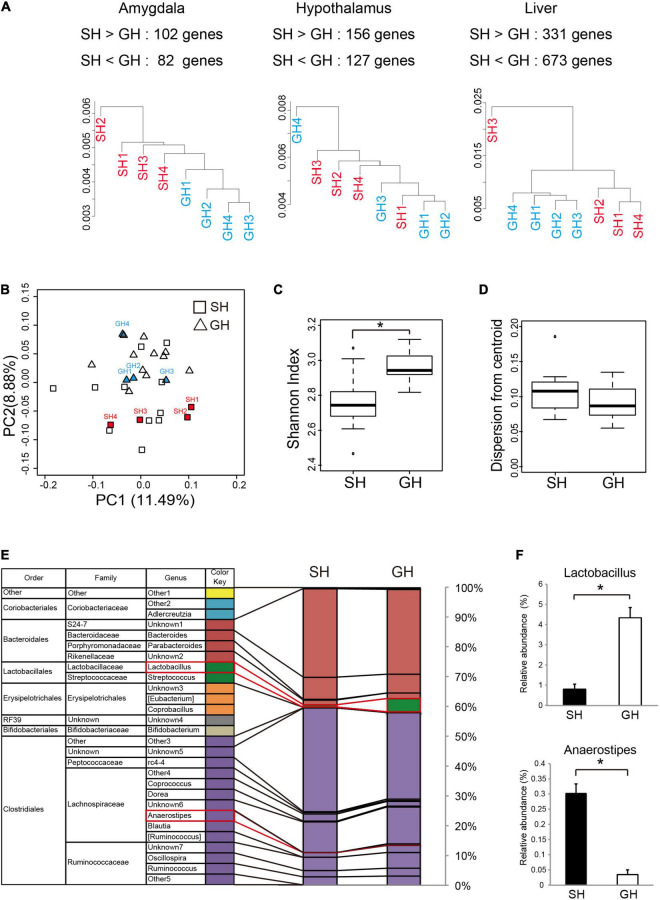
Effect of housing condition on the tissue transcriptome and the gut microbiome. **(A)** Cluster analysis of the genes expressed in the amygdala, the hypothalamus, or in the liver. Protein coding sequences with the expression levels of more than 0.3 reads per kilobase of exon per million (RPKM) mapped reads were analyzed. The number of differentially expressed genes (SH > GH or SH < GH) was calculated from the statistical comparison by DEseq2 (*n* = 4 for each group). **(B)** Principal coordinate analysis (PCoA based on Bray-Curtis distance) of the cecal microbiome at the genus level. Mice used for correlation analysis were marked by the abbreviation same as in **(A)** (*n* = 15 for each group). **(C)** Alpha-diversity analysis of cecal microbiome (*n* = 15 for each group). The Shannon index of the SH group was significantly lower than that of GH group, *p* = 2.40 × 10^– 4^. **(D)** The dispersion from each centroid was calculated from Bray-Curtis distance of each group. No significant difference was observed among the groups (*n* = 15 for each group). **(E)** The composition of cecal bacterial genera was analyzed by 16s rDNA amplicon sequencing (*n* = 15 for each group). Names in parenthesis were temporarily assigned to bacteria in the reference data set (Rainey and Janssen, 1995). **(F)** Significant between-group differences (*) were detected in the abundances of *Lactobacillus* (Lactobacillales, Lactobacillaceae, SH < GH, *p* = 8.98 × 10^– 4^), and *Anaerostipes* (Clostridiales, Lachnospiraceae, SH > GH, *p* = 2.42 × 10^– 4^). The color code for the bacteria species was represented at the genus level. *Significant differences were detected by unpaired *t*-test (*p* < 0.05).

To examine the kind of molecular change that underlies these biochemical and behavioral phenotypes, we analyzed the transcriptome of the amygdala, hypothalamus, and liver in the mice raised in SH condition. We chose these tissues according to their contribution to the perception of social stimuli by the brain and to the regulation of metabolic status (Tanaka et al., 2017, 2018). Especially in the social interaction context, stimuli from the other individuals seemed to be evaluated primarily by the amygdala, and the resulting emotional change of the brain may descend through the HPA axis to regulate metabolism. Cluster analysis of all gene expression revealed moderate segregation of individual transcriptomes into SH or GH branches (Figure 3A). The statistical analysis identified the DEGs in each tissue (SH < or > GH with numbers, Figure 3A). We also analyzed the cecal microbiome expecting its fluctuation by the change in inter-individual interaction. The cecum content of mice was analyzed by next-generation sequencing of bacterial 16S rRNA gene amplicon and analyzed at the genus level. In the PCoA, GH and SH samples were evenly distributed along the PC1 axis, while they were relatively separated along the PC2 (Figure 3B). There was a significant difference in the diversity among the individuals (Shannon index, Figure 3C), while no significant difference was detected in the diversity within the groups (dispersion from the centroid, Figure 3D). As implicated by the principal coordinate plot, there were significant differences in the abundance of the two bacterial genera, where the microbiome of the SH group contained less *Lactobacillus* (green) or more *Anaerostipes* (violet) than the GH group (Figures 3E,F).

### Regulation of Tissue Signals Under the Single Housing Condition

To elucidate cellular signals that regulate gene expression in the tissues, we analyzed the enrichment of DEGs in the gene sets regulated by known cellular or endocrine signals (Ingenuity Pathway Analysis, IPA). Significant enrichment of DEGs was observed in 10 terms in the amygdala, 5 terms in the hypothalamus, and 18 terms in the liver. In the amygdala (Table 1), FAS-associated death domain protein (FADD) and histone deacetylase (Hdac) signals were predicted to be activated in SH condition (positive z-scores). On the other hand, multiple pathways related to cAMP signaling, cAMP-responsive element modulator (CREM), cAMP-responsive element-binding protein 1 (CREB1), and cyclic-dependent protein kinase (Pka) showed suppression in SH condition (−2.75 < Z-score >−2.00). The other regulators included two transcription factors; forkhead box O3 (FOXO3) and nuclear receptor subfamily 3 group c member 2 (glucocorticoid receptor, NR3C2). In the hypothalamus (Table 2), a miRNA species involved in clock gene regulation (mir-21) (Brianza-Padilla et al., 2018), signal transducer and activator of transcription 5A/B (STAT5a/b), and Calmodulin were predicted to be activated in SH condition. There were two regulators that showed suppression in SH condition, Huntingtin (HTT) and protein kinase C [PKc(s)]. The term, PKc(s) was also detected in the amygdala as a suppressed regulator, whose downstream genes included Per1, Fos proto-oncogene (FOS), and albumin D-box binding protein (DBP) (Table 1). In the liver (Table 3), two regulators related to endocrine system (Prolactin and estrogen receptor), two cytokines [tumor necrosis factor (TNF) and interferon gamma (IFNG)], four factors related to metabolic regulation [PARP1 binding protein (PARPBP), nuclear receptor subfamily 0 group b member 2 (NR0B2 or small heterodimer partner), MLX interacting protein-like (MLXIPL or ChREBP), and nuclear factor, erythroid 2 like 2 (NFE2L2 or Nrf2)] were predicted to be activated in SH condition. There were ten suppressed regulators with various functions; secreted protein acidic and cysteine rich (SPARC), ten-eleven translocation methylcytosine dioxygenase 2 (TET2), caveolin 1 (CAV1), rapamycin-insensitive companion of mammalian target of rapamycin (RICTOR), aryl hydrocarbon receptor nuclear translocator (ARNTL), serine and arginine-rich splicing factor 2 (SRSF2), fatty acid-binding protein 2 (FABP2), insulin receptor substrate 1 (IRS1), and presenilin 1 and 2 (PSEN1 and 2).

**TABLE 2:** The functional enrichment analysis of the genes affected by single housing (SH) condition in the hypothalamus.

Tissue	Upstream Regulator	Description	Predicted Activation State	Activation z-score	*p*-value of overlap	Gene regulation	Bacteria correlated	No correlation
Hypothalamus	mir-21	miRNA	Activated	2.57	3.13.E-03	SH up		Smarca4
						SH down	Iigp1 (*Dorea*+), Per3 (*Lactobacillus*+)	Casp12, Dbp, Per2, Timp3
	STAT5a/b	Signal Transducer And Activator Of Transcription 5A/B	Activated	2.00	6.29.E-03	SH up		Stip1
						SH down	Rara (*Anaerostipes*−)	Cavin2, Mrvi1, Stip1, Vwf
	Calmodulin	Calmodulin	Activated	2.00	2.70.E-03	SH up		Calm1, Epha6, Rph3a, Syt1
						SH down		
	HTT	Huntingtin	Inhibited	−2.90	2.82.E-13	SH up	Fabp7 (*Anaerostipes*+)	Arpp19, Cck, Cit, Cplx2, Cx3cl1, Gad1, Hspa8, Kcnip2, Nefm, Nos1, Pcp4, Pde1b, Plcb1, Rph3a, Schip1, Snca, Tbr1
						SH down	Sfrp1 (*Dorea*+)	Abca1, Adamts2, Cnr2, Dbp, Fos, Per1, Per2, Rbp4, Spp1, Thbs2, Timp3
	Pkc(s)	Protein Kinase C	Inhibited	−2.20	4.33.E-03	SH up		Hsp90b1
						SH down		Abca1, Dbp, Fos, Per1, Per2

**TABLE 3:** The functional enrichment analysis of the genes affected by single housing (SH) condition in the liver.

Tissue	Upstream Regulator	Description	Predicted Activation State	Activation z-score	*p*-value of overlap	Gene regulation	Bacteria correlated	No correlation
Liver	TNF	Tumor Necrosis Factor	Activated	3.46	1.15.E-06	SH up	Abcb11 (*Lactobacillus*−), Alox8 (*Lactobacillus*−), Krt8 (*Anaerostipes*+), Kynu (*Lactobacillus*−, *Anaerostipes*+), Psmb9 (*Lactobacillus*−)	Actb, Apoc2, Bace1, C4b, Hc, Ccl6, Cdh1, Cyp7b1, Dbi, 8430408g22rik, Efna1, Enpp3, Fgg, Fst, Fth1, Il4ra, Krt18, Lamb3, Lgals9, Lpl, Nme1, Oas2, Oasl1, Pde4b, Pilrb2, Psme2, Rab32, Rbpms, Rps13, Rps15a-ps4, S100a10, Serpina3k, Sox9, Sqstm1, St3gal3, Stat3, Stat5a, Tnfaip2, Traf3
						SH down	Bhlhe40 (*Lactobacillus*+), Cyp2e1 (*Lactobacillus*+), Nr1h4 (Fxr) (*Lactobacillus*+), Pcyt1a (*Lactobacillus*+), Usp2 (*Lactobacillus*+)	Abcc2, Apoe, Atp1a1, Bcl2l11, Dpys, Dusp1, Enpp2, Il15, Iarid2, Maff, Pbrm1, Ppara, Prkca, Slc1a2, Slc20a1, Srebf1, St3gal5, 1810011o10rik, Timp3, Ugcg, Vegfa
	estrogen receptor	estrogen receptor	Activated	2.41	2.24.E-03	SH up	Krt8 (*Anaerostipes*+)	Cdh1, Col4a1, Erbb3, Hsp90aa1, Krt18, Ly6e, Pcdh1, Pdgfc, Tjp3
						SH down		Bmp7, Cav2, Ccne2, Fgf1, Klf10, Por, Tgfbr3, Timp3, Vegfa
	PRL	Prolactin	Activated	2.37	4.75.E-04	SH up	Dhx58 (*Clostridiales*_Unknown5+)	Actb, Cdh1, Erbb3, Helz2, Herc6, Ly6e, Oas2, P4hb, Pdk4, Pnpt1, Psme2, Rpsa, Serpina3k, Tmem140, Tmsb4x, Trim14
						SH down		Camk2n1, Cnn3, Ecm1, Igfals
	IFNG	Interferon Gamma	Activated	2.30	5.29.E-03	SH up	Abcb11 (*Lactobacillus*−), Psmb9 (*Lactobacillus*−)	Actb, Btg1, C4b, Fam26f, Ccl6, Ccna2, Celsr1, Clic4, Ctnnb1, Dapk1, Fth1, H6pd, Herc6, Ifi47, Irgm1, Irs2, Itpk1, Lpl, Ly6e, Ncald, Oas2, Oasl1, Psme2, Rarres1, Slfn1, Stat3
						SH down	Cyp2e1 (*Lactobacillus*+)	Agpat1, Aqp11, Atp1a1, Atp1b1, Bcl2l11, Bmf, Dusp1, Fech, Fgf1, Foxo1, Gck, Il15, P2ry1, Ppara, Rorc, Slc1a2, Slc6a6, Srebf1, Tgfbr3, Timp3, Vegfa
	NR0B2 (SHP)	Nuclear Receptor Subfamily 0 Group B Member 2 (small heterodimer partner)	Activated	2.29	1.23.E-04	SH up	Abcb11 (*Lactobacillus*−)	Cyp7b1, P4hb, Pdia4
						SH down	Cpt1a (*Lactobacillus* +), Nr1h4 (Fxr) (*Lactobacillus*+)	Acadm, Cyp7a1, Gck, Srebf1
	PARPBP	PARP1 Binding Protein	Activated	2.24	2.28.E-03	SH up	Krt8 (*Anaerostipes*+)	Krt18,
						SH down	Cyp2e1 (*Lactobacillus*+)	Amd1, Inmt
	MLXIPL (ChREBP)	MLX Interacting Protein Like (lipogenic transcription factor)	Activated	2.14	5.66.E-04	SH up		Acacb, Rpl15, Rpl27, Rpl7a, Rplp0, Rps13, Rps26, Rpsa
						SH down	Cpt1a (*Lactobacillus*+)	Hmgcr, Mid1ip1, Ppara, Srebf1, Thrsp
	NFE2L2 (nrf2)	Nuclear Factor, Erythroid 2 Like 2	Activated	2.10	1.22.E-08	SH up	Abcb11 (*Lactobacillus*−), Arhgef3 (*Oscillospira*−), Dhcr7 (*Lactobacillus*−), Ppib (*Lactobacillus*−), Prdx1 (*Anaerostipes*+)	Bhmt, Hc, Calm1, Cdh1, Cul1, Cyp4a12b, F10, Fth1, G6pdx, Gpx1, Hsp90aa1, Lpl, Ly6e, Mcfd2, Nucb2, Pdia4, Pdia6, Psmb6, Psmd14, Rplp0, Serpina3k, Srxn1, Ugt2b38
						SH down	Bnip3 (*Lactobacillus*+)	Alas1, Atp1a1, Foxo3, Ifrd1, Inmt, Ngef, Nr1i3 (Car), Ogt, Serpina6, Slc38a3, Srebf1, Thrsp, Vegfa
	SPARC	Secreted Protein Acidic And Cysteine Rich	Inhibited	−3.86	4.28.E-05	SH up		Cdh1, Cldn1, Mcm6, Mpzl1, Sox9, Trim2, Ube2u
						SH down	Bhlhe40 (*Lactobacillus*+), Usp2 (*Lactobacillus*+), Wnk4 (*Lactobacillus*+)	Cdyl, Ciart, Per2, Syde2, Tsku,
	RICTOR	RPTOR Independent Companion Of MTOR Complex 2	Inhibited	−3.55	3.78.E-04	SH up	Atp6v0a1 (*Anaerostipes*+), Psmb9 (*Lactobacillus*−)	Atp5l, Bsg, Fabp5, Irs2, Ndufc1, Oas2, Ppa1, Psmb6, Psmc4, Psmd14, Psme2, Rpl34, Rpl7a, Rplp0, Rps13, Rps26, Rpsa
						SH down		Ar, Bcl2l11, Gck, Prkca, Rorc, Srebf1
	PSEN2	Presenilin 2	Inhibited	−2.72	3.23.E-03	SH up	Prc1 (*Oscillospira*−), Psen2 (*Lactobacillus*−)	Actb, Cd9, Ctnnb1, Gapdh, Zfp101
						SH down	Dbp (*Lactobacillus*+)	Enpp2, Foxo3, Fzd4
	ARNTL	Aryl Hydrocarbon Receptor Nuclear Translocator Like	Inhibited	−2.60	1.18.E-03	SH up		
						SH down	Dbp (*Lactobacillus*+), Per3 (*Lactobacillus*+)	Gck, Per1, Per2, Ppargc1b, Srebf1
	TET2	Tet Methylcytosine Dioxygenase 2	Inhibited	−2.50	7.80.E-03	SH up		Ctnnb1, Grk5, Pxdc1, Rnf144a
						SH down	Bhlhe40 (*Lactobacillus*+)	Aspa, Bcl2l11, Hs6st1, Maff, Mtss1, Sh2b3, Slc16a12, Slc20a1
	SRSF2	Serine And Arginine Rich Splicing Factor 2	Inhibited	−2.43	4.80.E-04	SH up		Cdh1
						SH down	Nr1h4 (Fxr) (*Lactobacillus*+)	Ces1d, Nr1i3 (Car), Ppara, Srebf1
	FABP2 (IFABP)	Fatty Acid Binding Protein 2	Inhibited	−2.43	9.28.E-06	SH up		Acacb
						SH down	Cpt1a (*Lactobacillus*+)	Abcg5, Abcg8, Hmgcr, Ppara
	PSEN1	Presenilin 1	Inhibited	−2.32	1.15.E-04	SH up	Ank2 (*Lactobacillus*−), Prc1 (*Oscillospira*−), Tubb4b (*Lactobacillus*−)	Actb, Bace1, Bsg, Cd9, Ctnnb1, Dbi, Gapdh, Hsp90aa1, Nme1, Pgam1, Sqstm1, Stx1b, Tubb2a, Zfp101
						SH down	Dbp (*Lactobacillus*+), Per3 (*Lactobacillus*+), Pik3r1 (*Lactobacillus*+)	Apoe, Atp1b1, Dusp1, Enpp2, Foxo3, Fzd4, Mcm10, Hiat1, Pcp4l1, Rhoq, Slc1a2, Stim2, Tppp
	IRS1	Insulin Receptor Substrate 1	Inhibited	−2.05	1.10.E-03	SH up		Col4a1, Fst, Irs2, Lpl, Rhou
						SH down	Pik3r1 (*Lactobacillus*+)	Gck, Hmgcr, Ldlr, Ppap2b, Ppargc1b, Sparcl1, Srebf1, Vegfa
	CAV1	Caveolin 1	Inhibited	−2.04	3.76.E-03	SH up	Krt8 (*Anaerostipes*+), Tjp2 (*Lactobacillus*−)	Cdh1, Ctnnb1, Hsd17b12, Krt18, Nedd4l, Rps13,
						SH down	Npm1 (*Lactobacillus*+)	Cav2, Ccne1, Slc20a1

**FIGURE 4:**
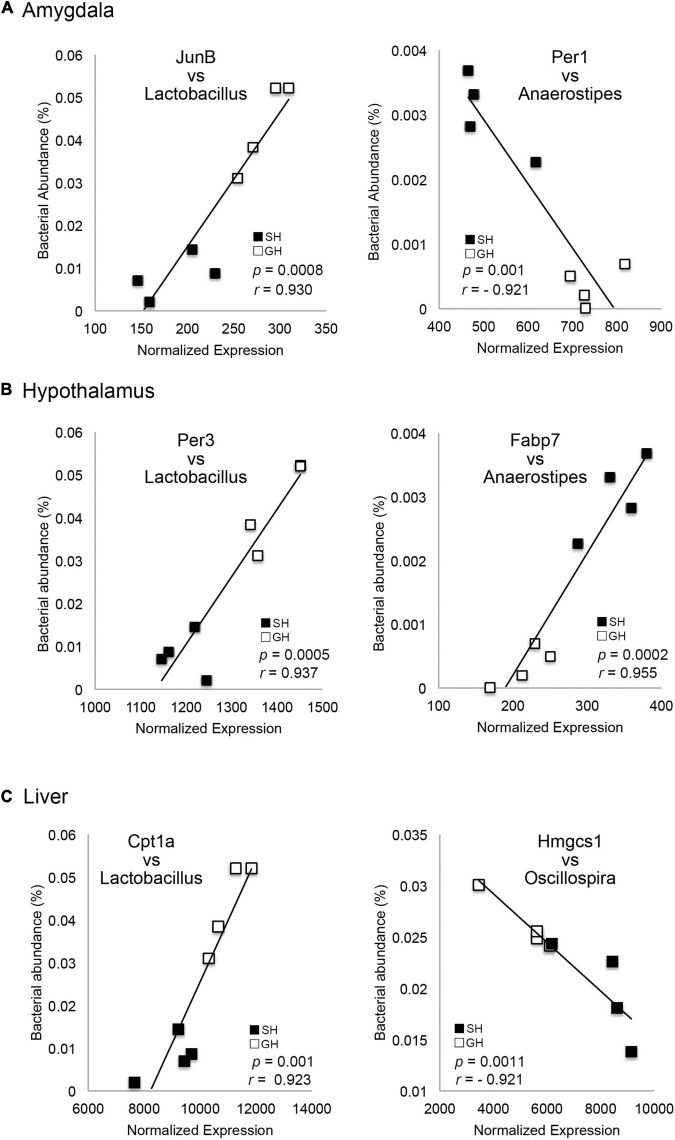
Correlation between the gene expression levels and the bacterial occupancy. The plot of each gene expression level in the amygdala **(A)**, hypothalamus **(B)**, or liver **(C)** against *Lactobacillus, Anaerostipes* or Oscillospira occupancy (*n* = 4 for each group). Genes were JunB Proto-Oncogene (JunB), Period 1 (Per1), Period 3 (Per3), Fatty acid-binding protein 7 (FABP7), Carnitine palmitoyltransferase 1A (CPT1A), and 3-Hydroxy-3-Methylglutaryl-CoA Synthase 1 (HMGCS1).

We then hypothesized that these changes in tissue transcriptomes could interact with the other homeostatic factors, namely, the gut microbiome, because recent studies have reported a significant correlation between host transcriptome and gut microbiome (Lim et al., 2020; Omura et al., 2020). The expression levels of DEGs in each tissue were normalized and their correlation with the occupancy of given bacteria genera was examined using Pearson’s correlation coefficient. Interestingly, there were multiple genes in the amygdala and hypothalamus that showed a significant correlation to the gut bacteria occupancy (Tables 1, 2 and Figure 4). Especially in the liver, there were a larger number of genes whose expression levels showed a significant correlation (Pearson’s correlation coefficient >0.92 and *p* < 0.01) to the abundance of two bacterial genera, *Lactobacillus* and *Anaerostipes*, in each animal (Table 3, Bacteria correlated). Correlation coefficients were both positive and negative, and some of them were represented in the plots; JunB Proto-Oncogene (JunB) vs. *Lactobacillus*, Period 1 (Per1) vs. *Anaerostipes*, Period 3 (Per3) vs. *Lactobacillus*, FABP7 vs. *Anaerostipes*, Carnitine palmitoyltransferase 1A (Cpt1a) vs. *Lactobacillus*, and 3-Hydroxy-3-methylglutaryl-CoA synthase 1 (Hmgcs1) vs. *Oscillospira*, as shown in Figure 4. The total numbers of bacteria-correlated genes were almost proportional to the DEGs in all tissues, 20 genes/182 genes in the amygdala, 35 genes/283 genes in the hypothalamus, and 151 genes/1,004 genes in the liver (Table 4). Some of them, such as kynureninase (KYUN) and cordon-bleu protein-like 1 (COBLL1), were inversely correlated to the abundances of *Lactobacillus* and *Anaerostipes*, respectively.

**TABLE 4:** Correlation between the expression levels of differentially expressed genes (DEGs) and the bacteria occupancy.

Tissue	Correlation	Direction	Other1	*Lactobacillus*	*Clostridiales* Unknown5	*Dorea*	*Lachnospiraceae* Unknown6	*Anaerostipes*	*Oscillospira*	Sum without redundancy
Amygdala	Positive	SH up	−	−	−	−	Fdft1	Cbwd1, Dcdc2a, Nrn1, Pdia3, Uqcrh	−	20 genes
		SH down	−	1700017B05Rik, Bhlhe41, Ccdc85b, Ier5l, Junb, Max	−	−	−	−	−	
	Negative	SH up	−	Mgea5, Nab1, Nrn1, St13	−	−	−	−	−	
		SH down	−	−	−	−	−	Faap100, Per1, Per3, Pkd1, Slc11a2	−	
Hypothalamus	Positive	SH up	−	−	−	−	−	Fabp7, Gm6793, Lhx9, Nr2f1, Nts, Rims1, Ruvbl2, Trnp1	−	35 genes
		SH down	−	Acox3, Angptl1, Hlf, Irx5, Nt5dc2, Per3, Slc2a4, Sspn, Stom, Wdr6	−	Bmp7, Cped1, Ddr2, Efemp1, Iigp1, Mxra8, Sfrp1, Slc16a12, Vasn	−	−	−	
	Negative	SH up	Mrrf	Cplx1, Epb4.1l1	−	−	−	−	Chd3, Map1a	
		SH down	−	−	−	−	−	Ptpn21, Rara, Sptlc2	−	
Liver	Positive	SH up	−	−	Cyp4a12a, Dhx58, Nat10, Prdx1	−	−	Abhd5, Atp6v0a1, AU022252, Caprin1, Cdcp1, Cgn, Clpx, Ddi2, Entpd8, Fbxo6, Fpgs, Gtpbp3, Insig2, Krt8, Kynu, Leprotl1, Mir8093, Nelfa, Pi4k2a, Pnkd, Pxmp4, Rbm45, Sphk2, St5, Tars, Traf3ip2, Ugt2b37	−	151 genes
		SH down	Mia2	Acot4, Aifm2, Ankrd12, Arhgap21, BC005537, Bhlhe40, Bnip3, Ccny, Chp1, Cobll1, Cpeb1, Cpt1a, Cyp2a5, Cyp2e1, D17Wsu92e, Dbp, Eci2, Eif4b, Ergic2, Fads2, Fam120b, Fam76a, Gm15787, Gm19619, Gpd1l, Hook2, Ipo5, Jph1, Kctd2, Klf13, Lonrf1, Mia3, Naa50, Nampt, Narf, Npm1, Nptn, Nr1h4, Osbpl1a, Pcgf5, Pcyt1a, Pde8a, Per3, Phf7, Pik3r1, Rap2a, Rapgef1, Rassf7, Rragc, Slc5a6, Slmap, Sox12, Ssfa2, Swap70, Tbc1d16, Timm9, Ttc7, Ttll5, Ubac1, Usp2, Uvrag, Vps13a, Vps26a, Wdr43, Wnk4, Zbtb7a, Zc3h8, Zfp91Cntf	−	−	−	−	1810055G02Rik, Pptc7	
	Negative	SH up	Tmem263	Abcb11, Alox8, Ank2, Apoc4apoc2, Avpr1a, Caprin1, Cdcp1, Cgn, Dhcr7, Dusp19, Faf2, Fam114a1, Fermt2, Gldc, Hsbp1l1, Htt, Kynu, Myh10, Net1, Pde9a, Ppib, Psen2, Psmb9, Rexo2, Rfwd3, Rnf141, Serpinf2, Sorbs2, Stard13, Stra6l, Tjp2, Traf3ip2, Tubb4b, Ubqln1, Zc3hav1	−	−	−	−	Arhgef3, Hmgcs1, Prc1, Sesn3	
		SH down	−	−	Phf14	−	−	2700081O15Rik, Acap2, AI464131, Ankrd12, Atxn7, Bud13, Cobll1, Dcun1d1, Dlgap4, Gm19619, Gtf2ird2, Hnrnpd, Lcorl, Narf, Nup50, S1pr5, Zfp385b	−	

*Genes in red and blue colors showed correlation to both Lactobacillus and Anaerostipes but in opposite way (SH up or down).*

**FIGURE 5:**
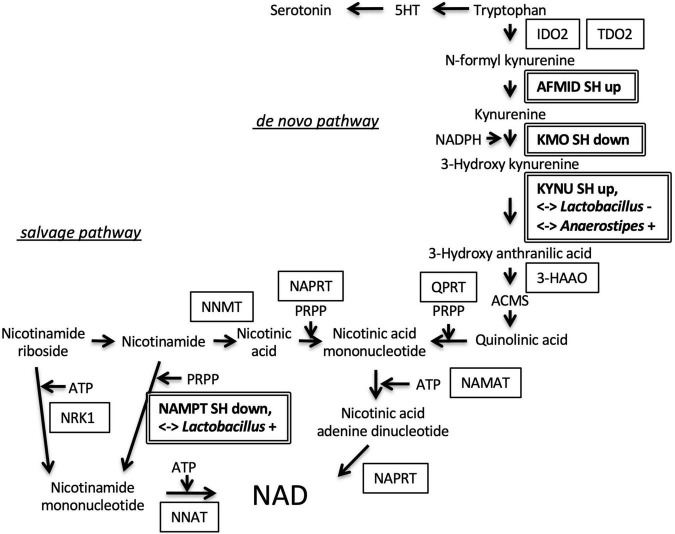
Regulation of genes involved in nicotinamide adenine dinucleotide (NAD) synthesis. Arylformamidase (AFMID) gene upregulated in SH condition and kynurenine 3-monooxygenase (KMO) gene downregulated in SH condition were located at the second, and third step of *de novo* nicotinamide NAD synthesis from tryptophan, respectively. The fourth enzyme, kynureninase (KYNU) gene was upregulated in SH condition and correlated with the occupancies of two bacteria genera (*Lactobacillus* and *Anaerostipes*). Nicotinamide phosphoribosyltransferase (NAMPT) gene was downregulated in SH condition and positively correlated with *Lactobacillus* occupancy. SH up, upregulated in SH condition; SH down, downregulated in SH condition; < – > *Lactobacillus* + or –, positively or negatively correlated with *Lactobacillus*; < – > *Anaerostipes* +, positively correlated with *Anaerostipes*; indoleamine 2, 3-dioxygenase 2, IDO2; tryptophan 2, 3-dioxygenase, TDO2; 3-hydroxyanthranilic acid 3, 4-dioxygenase, 3-HAAO; 2-amino-3-carboxymuconate-6-semialdehyde, ACMS; quinolinate phosphoribosyltransferase, QPRT; nicotinic acid mononucleotide adenylyl transferase, NAMAT; nicotinate phosphoribosyltransferase, NAPRT; nicotinamide N-methyltransferase, NNMT; nicotinamide riboside kinase 1, NRK1; nicotinamide nucleotide adenylyltransferase 1, NNAT; 5-phospho-D-ribose 1-diphosphate, PRPP.

We also examined the roles of DEGs in a specific metabolic pathway that might be located at the interface between the host endocrine system and gut microbiome. Interestingly, 4 enzyme genes were seemed to be enriched in the nicotinamide adenine dinucleotide (NAD) metabolizing pathway (Figure 5). Arylformamidase (AFMID), kynurenine 3-monooxygenase (KMO), and KYNU were located at *de novo* NAD synthesis pathway. Nicotinamide phosphoribosyltransferase (NAMPT) was located at the salvage pathway. Especially, the expression levels of KYNU and NAMPT genes were significantly correlated with two bacteria genera, *Lactobacillus* and *Anaerostipes* (Table 4).

### Suppression of Cyclic Adenine Monophosphate Signaling in the Amygdala by the Single Housing Condition

**FIGURE 6:**
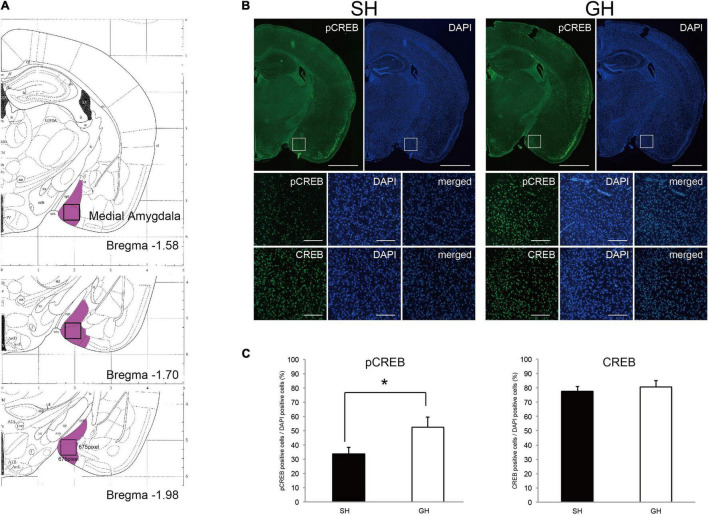
Immunohistochemical detection of cyclicAMP signaling in the medial amygdala of single housing (SH) and group housing (GH) mice. **(A)** Region of interest (ROI, open box) in the medial amygdala (violet area, ref. Paxinos and Franklin’s the Mouse Brain in Stereotaxic Coordinates). **(B)** Immunostaining of the coronal sections using anti-phospho cyclic AMP-responsive element-binding protein (pCREB) or anti-CREB antibody. Scales: 500 micrometers for the first-row panels and 50 micrometers for the second and the third row panels. **(C)** Quantification of anti-pCREB and anti-CREB positive nuclei (mean ± SEM, 4 sections for each mouse, *n* = 3 or 4). *Significant differences were detected by unpaired *t*-test (*p* < 0.05).

Given that cAMP signaling was expected to be suppressed in the amygdala of SH mice, we used immunohistochemistry to detect this signal at the cellular level. Since the medial amygdala plays a pivotal role in social recognition, we examined the phosphorylation status of CREB in this ROI (Figure 6A). Two antibodies; anti-CREB antibody that recognizes both phospho- and non-phospho forms and anti-pCREB antibody that recognizes phosphorylated form alone, were used. We compared the fraction of immunoreactive nuclei number per DAPI-stained nuclei number in ROI (Figure 6B). As a result, significant differences in the fraction of pCREB-positive nuclei were observed between the SH and GH groups but not in the fraction of CREB-positive nuclei (Figure 6C).

## Discussion

**FIGURE 7:**
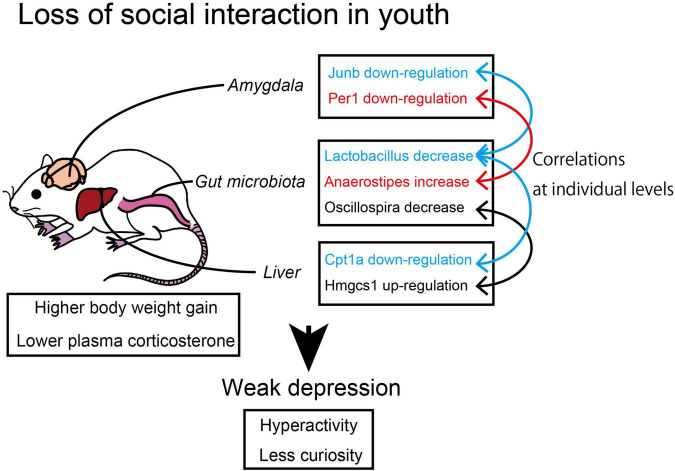
The correlation between microbiome and transcriptome in socially isolated mice. The young mice raised under single housing (SH) condition showed suppressed cyclic adenine monophosphate (cAMP) signaling in the amygdala and suppressed lipolysis in the liver with the coordination of gut bacteria genera.

In this study, we found that the short-term social isolation of animals induced weak depression-like behavior accompanied by the suppression of the cAMP signaling in the amygdala. In addition, some of the DEGs in the amygdala, hypothalamus, and liver showed a significant correlation to the abundance of the two gut bacterial genera, *Lactobacillus* and *Anaerostipes*. The results suggested that the environmental factor of SH conditions along with the coordinated change in the gut bacteria composition affected the behavior and metabolism of animals (Figure 7).

It has been reported that the mature mice raised in SH conditions over the long term (4–6 weeks) show depression-like behavior (Skelly et al., 2015; Ieraci et al., 2016). Our housing period (2 weeks) was shorter than these conditions, but our SH mice still showed weak depression-like behavior and lower plasma corticosterone levels. These results suggest a strong correlation between the depressive phenotypes and the lower basal corticosterone levels, which, in turn, can affect body metabolism. In our case, SH mice showed higher body weight and higher assimilation ratio than GH mice (Figures 1C,D). This could be caused by a reduction in the basal metabolic rate as observed with the rats exposed to prenatal stress (Iturra-Mena et al., 2018). Considering that the moving area per one mouse was wider in SH conditions (198 cm/1 mouse) than in GH conditions (413 cm/5 mice = 82.6 cm/1 mouse), a higher assimilation ratio of SH cannot be simply attributable to the moving activities of mice. Because corticosterone serves as a catabolic hormone in normal conditions, lower level of corticosterone observed in SH mice may account for a higher assimilation quotient (lower catabolism) of SH mice (Macfarlane et al., 2008).

It is possible that these behavioral and metabolic phenotypes arise from some change in information processing in the brain of SH mice. Amygdala may be the most responsible region for social behavior because it is a center for evaluation and emotion responses (Ferretti et al., 2019; Harada et al., 2019; Reisinger et al., 2019). Based on the results of the transcriptome analysis, we predicted the suppression of the cAMP signaling in the amygdala of SH mice, which was confirmed by the lower level of CREB phosphorylation observed in the medial amygdala (Figures 6B,C). There are emerging pieces of evidence that activation of the cAMP signaling in the amygdala facilitates the consolidation of social memory (Viosca et al., 2009; Tronson et al., 2012; Cowansage et al., 2013; Ranjan et al., 2015; Hamidkhaniha et al., 2019). Accordingly, the impaired memory and social interaction observed in SH mice could be attributable to the repression of the cAMP signaling in the amygdala. As to the other part of the brain, it is notable that the term HTT was identified as an activated regulator in the hypothalamus (Table 2). Because the transgenic mice that express mutated HTT protein have been shown to exhibit depression, such as behavior, it is possible that HTT-related signaling may be responsible for the progression of depression symptom (Hult Lundh et al., 2013). It is not clear how these brain signals are involved in the HPA axis that governs the corticosterone production. However, we detected suppression of mineral corticoid receptor (NR3C2) signal in the amygdala. NR3C2, along with glucocorticoid receptor (NR3C1), constitutes a feedback loop in the HPA axis (Keller et al., 2017). Further study is needed to elucidate the connection among cAMP, NR3C2, and HTT signals in the brain.

The regulation of lipid metabolism in the liver of SH mice can be characterized by several enzyme genes, such as Hmgcr (SH down), Cpt1A (SH down), Dhcr7 (SH up), Hmgcs1 (SH up), and Cyp7B1 (SH up). Among these, Hmgcr, Dhcr7, and Hmgcs1 positively contribute to steroidogenesis and the others contribute negatively. These regulations were not uniformly directed to corticosterone production in SH condition as indicated by their regulation opposing to each other. In addition, we did not detect a significant change in TG or cholesterol content in the liver of SH mice. Accordingly, we conclude that these gene regulations may not be drastic enough to affect the corticosterone levels at the level of raw material supply. Nevertheless, SH mice showed a significant higher body weight than GH mice. Part of the reason for this phenotype can be the decreased expression of Cpt1A, which may lead to the lower lipid catabolism in SH mice (Do et al., 2011; Nyman et al., 2011).

There are several potential environmental factors that might have induced changes in the brain and the liver homeostasis observed in this study. We speculate that the environmental factors for young animals can be classified into two types; biophysical or chemical stimuli received by the sensory system and microbiological interactions. It is understood that the SH mice received less sensory stimuli from the other individuals than the GH mice. Human studies have shown that social isolation causes a change in the activity of the amygdala (Bolling et al., 2011; Bickart et al., 2012; Von Der Heide et al., 2014; Tian et al., 2015). Another possibility could be the microbiological influences, which are exerted by the SH condition, which inhibits inter-animal exchange of gut microbiota through feces; this may eventually inhibit the diversity of microbiota in individual animals. However, our results did not support this possibility since we observed a significant difference in the alpha diversity within the individuals (Shannon index) but not in the beta diversity (the dispersion from the centroid of each group, Figures 3C,D). Lundberg et al. (2017) studied inter-individual interaction in mice with special reference to the gut microbiota. They maintained the mice for 7 weeks in individually ventilated cages where they were prevented from having microbiological and sensory interactions. They found a difference in the alpha diversity between the SH and GH groups (Richness and Shannon indices) but not in the beta diversity among the groups. These results indicate that the reduction in inter-individual bacterial exchange does not influence the inter-individual bacterial diversity. More importantly, we observed an overall segregation of microbiome between the SH and GH conditions as indicated by the PCoA and by the differences in the abundance of two bacterial genera, *Lactobacillus* and *Anaerostipes* (Figure 3D). Taken together, it is possible that the decrease in social sensory stimuli could be the primary environmental factor in the SH condition.

It is notable that the multiple DEGs in the amygdala, hypothalamus, and the liver exhibited a significant correlation to the abundance of multiple bacterial genera, such as *Lactobacillus* and *Anaerostipes* (Tables 1–4 and Figure 4). The percentage of these bacteria-correlated DEGs was about 15%, suggesting the possibility that the coordination of gene expression by the bacteria was limited and the majority of the genes was more directly regulated by the environmental condition. Conversely, four of the bacteria (Other 1, Unknown 5, Dorea, and Unknown 6) did not show any significant difference in their occupancies between the experimental groups (Supplementary Table 1). This may have arisen from their difference in the sensitivity to the individual fluctuation of homeostasis. The bacteria-correlated DEGs included JunB in the amygdala, Period genes (Per1, 3) in the three tissues, and Cpt1A in the liver. There are increasing reports about the metabolic influence of short-chain fatty acids produced by the bacterial species (e.g., acetate by *Lactobacillus* and butyrate by *Anaerostipes*) on their hosts. Whether these products are directly linked to the metabolism of the host is yet to be confirmed but our results suggested that a significant number of host genes could be affected by bacteria (Supplementary Table 1). It is possible that some bacteria play essential roles in coordinating brain and tissue homeostasis in response to the social input. For example, Xie et al. (2019) reported an ameliorating effect of *Lactobacillus reuteri* on depressive behavior and its correlation to serotonin, whose precursor, tryptophan can be metabolized by Kynu and Nampt into NAD. More importantly, Scarborough et al. (2021) investigated the role of serotonin in the social isolation model using serotonin reuptake inhibitor, fluoxetine (FLX). They found that FLX treatment was effective to prevent anxiety-related behaviors both in the isolated generation (dams) and in their offspring. These results suggested that NAD precursors may be located at the interface between the host and microbiome during the progression of depression. In this study, we found that KYUN was upregulated in the liver of SH mice and showed a correlation to the occupancies of *Lactobacillus* (negative) and *Anaerostipes* (positive) while NAMPT was downregulated and positively correlated to *Lactobacillus* (Table 4). The upregulation of KYNU may facilitate *de novo* NAD synthesis from tryptophan while the downregulation of Nampt may suppress a salvage circuit from nicotinamide mononucleotide (Figure 5). Rudzki et al. (2019) reported that supplementation of *Lactobacillus* to the patients with depression decreases kynurenine concentration and improves cognitive functions. It is important to examine whether these NAD-related substances are responsible for the weak depression phenotype or not, with the help of prebiotics, such as guar gum (Shats et al., 2020; Chen et al., 2021). Our results may shed light on the metabolic regulation by the bacterial coordination during the progression and the healing process of weak depression. The SH responsive genes identified in the three tissues and SH responsive gut bacteria will be useful in identifying and developing food-based therapeutics for depression.

## Data Availability Statement

The data presented in the study are deposited in the Gene Expression Omnibus repository, accession number GSE198597.

## Ethics Statement

The animal study was reviewed and approved by animal committee of the Innovation Center of NanoMedicine (Kawasaki, Kanagawa, Japan).

## Author Contributions

KS, MN, AK, FS, KK, RI, AY, TK, and KA designed and planned the experiments. KS, MN, and AY performed the experiments. KS, MN, RI, AY, and TK analyzed the data, created the figures, and wrote the manuscript. All authors reviewed and approved the final manuscript.

## Conflict of Interest

The authors declare that the research was conducted in the absence of any commercial or financial relationships that could be construed as a potential conflict of interest.

## Publisher’s Note

All claims expressed in this article are solely those of the authors and do not necessarily represent those of their affiliated organizations, or those of the publisher, the editors and the reviewers. Any product that may be evaluated in this article, or claim that may be made by its manufacturer, is not guaranteed or endorsed by the publisher.

## Footnotes

1
https://www.ncbi.nlm.nih.gov/assembly/GCF_000001635.20/

2
https://sourceforge.net/projects/subread/files/subread-2.0.0/

3
https://bioconductor.org/packages/release/bioc/html/DESeq2.html

4
https://www.rdocumentation.org/packages/stats/versions/3.6.2/topics/hclust

5
www.qiagen.com/ingenuity

6
https://gold.jgi.doe.gov

7
https://www.drive5.com/usearch/

8
http://www.skylight-biotech.com

## Supplementary Material

Supplementary Figure 1 Fundamental data of behavioral tests. **(A)** Definition of Social zone, Neutral zone, and their subdivided areas. **(B)** Stopping time in each subdivided area. A significant difference was detected only when the values were summed up within the Social or Neutral area (Figure 2A). **(C)** The objects used in the Novel object recognition test. **(D,E)** Size of Y-maze and open field.

## Abbreviations

cAMP: cyclic adenosine monophosphate
DEGs: differentially expressed genes
GH: group housing
NAD: nicotinamide adenine dinucleotide
PCoA: principal coordinate analysis
RPKM: the reads per kilobase of exon model per million mapped reads
SH: single housing.
