# Supplementary figures and images for: Mouse Model of Weak Depression Exhibiting Suppressed cAMP Signaling in the Amygdala, Lower Lipid Catabolism in Liver, and Correlated Gut Microbiota
Source: Front Behav Neurosci. 2022 May 19;16:841450. doi: 10.3389/fnbeh.2022.841450 (PMC9345170; doi:10.3389/fnbeh.2022.841450)

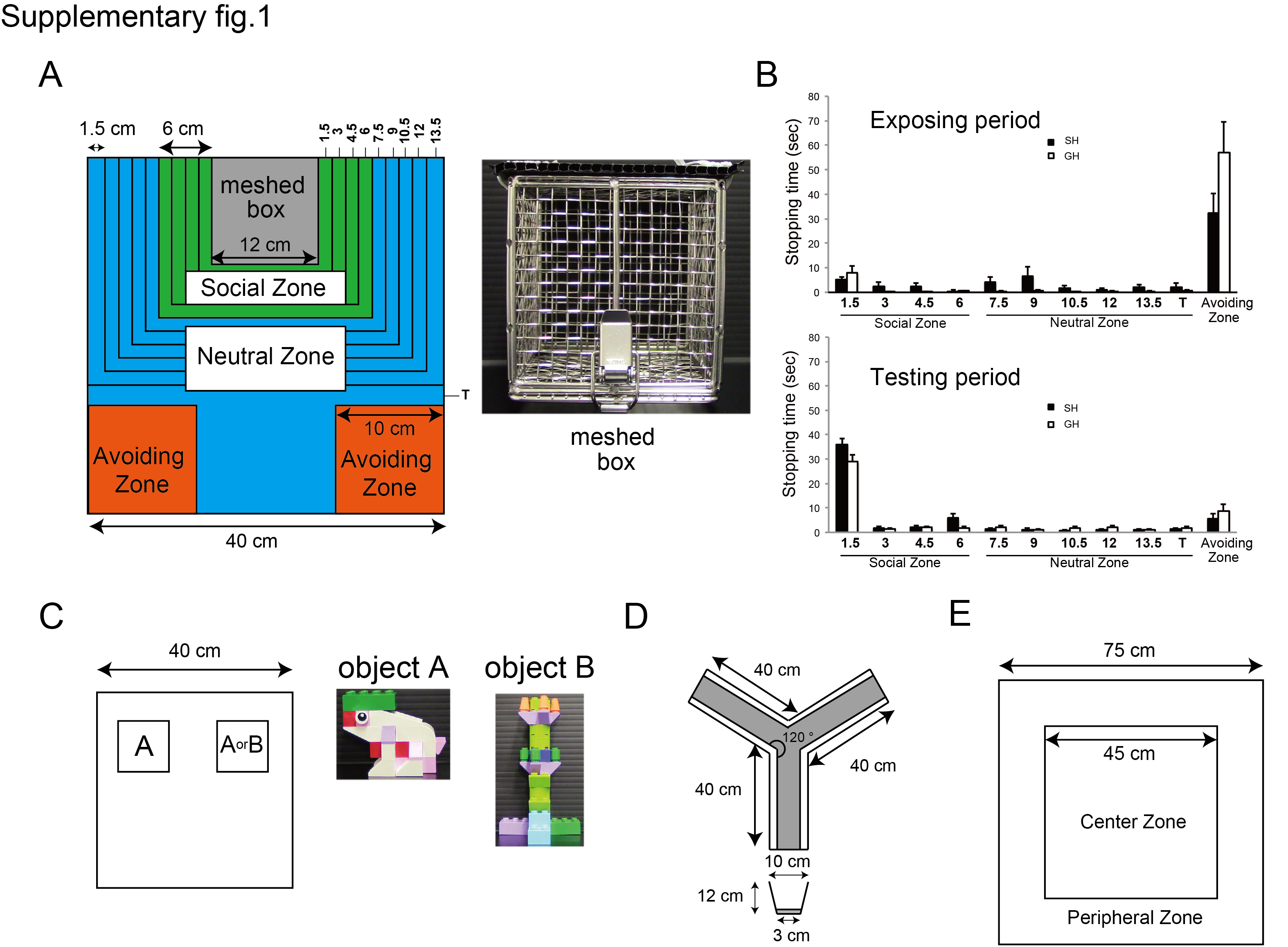

Supplement: Supplementary Figure 1 — Fundamental data of behavioral tests. (A) Definition of Social zone, Neutral zone, and their subdivided areas. (B) Stopping time in each subdivided area. A significant difference was detected only when the values were summed up within the Social or Neutral area (Figure 2A). (C) The objects used in the Novel object recognition test. (D,E) Size of Y-maze and open field. [file Image_1.JPEG]
