# Supplementary material for: Mouse Model of Weak Depression Exhibiting Suppressed cAMP Signaling in the Amygdala, Lower Lipid Catabolism in Liver, and Correlated Gut Microbiota
Source: Front Behav Neurosci. 2022 May 19;16:841450. doi: 10.3389/fnbeh.2022.841450 (PMC9345170; doi:10.3389/fnbeh.2022.841450)
Supplement: Supplementary file 2 [file Table_1.DOCX]

| Order | Family | Genus | Average±SEM | | T test *p-*value |
| --- | --- | --- | --- | --- | --- |
|  |  |  | Group (n=15) | Single (n=15) |  |
| Other | Other | Other1 | 0.20±0.02 | 0.14±0.02 | 0.08 |
| *Coriobacteriales* | *Coriobacteriaceae* | Other2 | 0.10±0.02 | 0.08±0.02 | 0.58 |
|  |  | *Adlercreutzia* | 0.15±0.02 | 0.11±0.01 | 0.14 |
| *Bacteroidales* | S24-7 | Unknown1 | 27.2±0.99 | 30.0±1.19 | 0.08 |
|  | *Bacteroidaceae* | *Bacteroides* | 7.08±0.77 | 7.38±0.88 | 0.80 |
|  | *Porphyromonadaceae* | *Parabacteroides* | 0.10±0.02 | 0.25±0.05 | 0.02 |
|  | *Rikenellaceae* | Unknown2 | 1.69±0.14 | 1.32±0.35 | 0.34 |
| *Lactobacillales* | *Lactobacillaceae* | *Lactobacillus* | 6.81±0.72 | 3.30±0.79 | **2.78.E-03** |
|  | *Streptococcaceae* | *Streptococcus* | 0.04±0.01 | 0.03±0.01 | 0.46 |
| *Erysipelotrichales* | *Erysipelotrichales* | Unknown3 | 0.09±0.02 | 0.13±0.04 | 0.36 |
|  |  | *[Eubacterium]* | 0.01±4.79.E-03 | 0.02±0.01 | 0.27 |
|  |  | *Coprobacillus* | 0.01±4.47.E-03 | 0.04±0.02 | 0.10 |
| RF39 | Unknown | Unknown4 | 0.02±0.01 | 0.09±0.07 | 0.34 |
| *Bifidobacteriales* | *Bifidobacteriaceae* | *Bifidobacterium* | 0.02±0.01 | 0.01±0.01 | 0.83 |
| *Clostridiales* | Other | Other3 | 0.02±0.01 | 0.01±3.41.E-03 | 0.93 |
|  | Unknown | Unknown5 | 29.6±0.80 | 31.9±0.78 | 0.05 |
|  | *Peptococcaceae* | rc4-4 | 0.36±0.11 | 0.26±0.08 | 0.48 |
|  | *Lachnospiraceae* | Other4 | 0.28±0.05 | 0.21±0.05 | 0.35 |
|  |  | *Coprococcus* | 2.29±0.24 | 2.06±0.23 | 0.50 |
|  |  | *Dorea* | 0.25±0.04 | 0.19±0.04 | 0.30 |
|  |  | Unknown6 | 9.58±0.47 | 10.0±0.79 | 0.65 |
|  |  | *Anaerostipes* | 0.08±0.02 | 0.25±0.03 | **9.23.E-05** |
|  |  | *Blautia* | 0.03±0.02 | 0.08±0.07 | 0.50 |
|  |  | *[Ruminococcus]* | 1.75±0.23 | 2.53±0.26 | 0.03 |
|  | *Ruminococcaceae* | Unknown7 | 4.63±0.38 | 3.90±0.40 | 0.19 |
|  |  | *Oscillospira* | 2.49±0.11 | 1.87±0.11 | **4.38.E-04** |
|  |  | *Ruminococcus* | 2.48±0.25 | 2.63±0.24 | 0.67 |
|  |  | Other5 | 0.07±0.01 | 0.09±0.02 | 0.41 |

**Supplementary Table 1 Occupancies of bacteria in each experimental group**

Bacteria showed significant correlation to the gene expression levels were shown in shaded columns. p-values lower than 0.01 were shown in bold letters. Names in parenthesis were temporary assigned to bacteria in the reference data set.
